# Strategic science translation in emerging science: genetically modified crops and Bisphenol A in two cases of contested animal toxicity studies

**DOI:** 10.1080/21645698.2022.2103368

**Published:** 2022-08-09

**Authors:** Monica Racovita, Armin Spök

**Affiliations:** Science, Technology and Society Unit, Graz University of Technology, Graz, Austria

**Keywords:** translation of science, contested science, science policy, risk governance

## Abstract

When controversies develop around scientific facts or technologies, the potential of science to become a tool in plays of interests and power between different actors is not well recognized. Cordner’s concept of Strategic Science Translation (SST) shows that such actions are enabled by the uncertainty and the complexity of the scientific processes that allow the use of science in support of various, often contradictory interests and goals. Two high-profile controversies around animal toxicity studies in two different fields of European regulatory science (genetically modified food and food contact materials) were chosen as case studies to explore and expand the SST concept. Both studies involve emerging science issues, emphasizing tensions between regulatory and academic science. Communications from key Civil Society Organizations (CSOs) and industry groups were used for analysis of each controversy. We found that both groups of actors try to present their own interpretation of scientific results, taking advantage of the lack of scientific consensus, of the uncertainties associated with the negotiation in the interpretation of results, and of the wider scientific and political context. In the same time, each actor attempts to challenge the credibility of the other. The lack of formal acknowledgment of the limitations of the emerging scientific fields, as well as of different research approaches between regulatory and academic research contribute to the continuation of controversies in the public domain, as the public cannot easily assess the information presented.

## 1. Introduction

The idea that science can act as a tool for different interests is not new. It has been shown that science can be strategically used and even manipulated by scientific advisors to influence policy decisions.^2,3^ Through this “politicization of science,” various institutional actors sometimes adopt an adversarial style toward each other, each claiming that they have science on their side, and ignoring their own normative assumptions. Furthermore, discussions about what constitutes evidence as a base for policy making go beyond science, affecting virtually all fields of government policy.^4,5^

As Eden^6^ indicated, interest groups, especially businesses, can use (and potentially distort) science to push policy in desired directions. They can also present their version of scientific facts, while insisting that policy should only be based on what they portray as ‘sound science.’ Although this phrase seems to indicate evidence-based policy, ‘sound science’ was first used by the tobacco industry as a public relations strategy to perpetrate doubt over cigarettes causing cancer.^7^

To advance their position, by claiming that it is supported by “sound science,” interest groups can also use data published in predatory journals. Published with minimal or without peer-review and quality checks, articles included in these journals are providing evidence of unclear and sometimes little quality while still pretending to scientific robustness.^8^ The business model of predatory journals has been very successful, with estimates of 15,000 predatory journals in 2020. In addition, their success forces traditional journals to increase the number of pages published, to stay competitive, thus allowing the publication of articles of slightly lower quality.^9^ These practices and trends have the potential to undermined scientific standards and provide an even broader range of studies portrayed as scientific and considered credible for strategic use in policy contexts.

While goal-oriented interpretation and communication of scientific information has been discussed in relation to groups with major economic interests, like the industry,^7,10^ little attention was given to other stakeholder groups. The concept of Strategic Science Translation (SST) provides a useful framework for analyzing, interpreting, and understanding the interpretation or reinterpretation of scientific data in the pursuit of different interests by all groups of stakeholders.^1,11^ SST shows that such actions are facilitated by the complexity and associated uncertainties of the scientific processes that allow the use of science in support of various, often contradictory interests and goals. “Strategic” in SST goes beyond selectivity in terms of data used, to include deliberate and manipulative behavior in obtaining and securing positions of power. It uses “coercion, resistance, dissimulation, and (de)legitimization” as well as “more benign acts of knowledge translation such as selective citation.”^1^ According to the concept of SST, science becomes a tool to support arguments in fields such as science policy or environmental activism.^1^ “Translation” too is done to support an argument, not simply to explain scientific jargon to nonscientific audiences. Cordner argues that all stakeholders involved with a particular technology or scientific field can use SST to strengthen their authority or determine change. They do so by employing three types of SST, often intermingled with each other: selective (“cherry-picking” studies to support a particular position or argument); interpretive (deliberatively highlight one aspect, while ignoring another; describe findings in a way that supports a particular stance); or inaccurate (assign incorrect findings or quality to a study).^1^ Cordner established the concept in the course of a large empirical study of the US controversy on flame-retardants. To date, the concept was used also referred to when explaining controversies in other contested policy arenas of environmental governance.^12,13^

For studies conducted in, or related to, various regulatory science contexts, like the animal toxicity studies analyzed in this article, controversies typically go beyond scientists, or directly invested stakeholders (like industry or advocacy groups), to sometimes even engulf the public at large. Such controversies often seem complex, persistent, and difficult to resolve. Despite the academic evidence placing values at the heart of controversies and policy research, official policy responses typically focus on scientific and technical aspects,^14,15^ suggesting that a major contribution to refueling controversies is the disregard for wider nonscientific issues. Yet when considering the relationship between scientific and nonscientific issues in the attempt to provide solutions for such controversies, the potential of science to become a tool in plays of interests and power between different actors is not well recognized.

In this paper, we employ and expand Cordner’s concept of SST^1,11^ to follow how actors “reinterpret” science to serve their interests in two controversies involving animal toxicity studies and their potential relevance for risk assessment in the regulatory context: a two-year rat feeding study in the field of genetically modified (GM) food and an animal study on Bisphenol-A (BPA), a substance widely used in food packaging. Both studies triggered high-profile controversies in the EU. The analysis will focus on industry and CSOs central to the respective controversies.

## 2. Contested Science

Science and technology studies have long ascertained that controversies around scientific issues are more than protracted disagreements on the content of knowledge or on the method employed to reach such content, and they include values, norms, political boundaries and cultural assumptions.^16^ Power claims can also be an integral part of the controversy.^17^ The interweaving of scientific, policy, ethical, and cultural factors means that debates presumable over scientific issues can acquire different dynamics and impede the achievement of a true closure.^18^

In addition to action by various stakeholders, uncertainties inevitably linked to scientific knowledge contributes to the appearance of controversies. Scientific uncertainties, understood as both limitations of the research process and the resulting scientific knowledge are ubiquitous in emerging and policy-relevant areas of research.^19^ These uncertainties can lead to provisional and inconclusive interpretations, which – if recognized as such – may be perceived by decision-makers as conflicting with the needs for indisputable references in decision-making processes. However, the necessity for accountability required that decision making be defendable. Science stepped in providing what was considered by decision-makers as the necessary and sufficient but also immutable evidence. This proved problematic in two ways. In the first, researchers from the history of science have long sustained that science does not produce instances of irrefutable proof but builds provisional consensus continuously subjected to scrutiny, reproducibility and revision, especially evident in emerging science.^20,21^ In the second, the immutability of evidence has been increasingly questioned, ironically, by policy makers themselves. As Blanchemanche et al. attest,^22^ pressure increased on scientists starting in the 1990s to indicate not just the extent of their knowledge but also their limitations. Thus, tensions arose between the portrayal of science in decision-making as providing irrefutable proof and increasing attention given to scientific uncertainties.

Uncertainties cannot be eliminated from scientific research and the resulting scientific knowledge. Even if very high certainty of safety is achieved in scientific risk assessment, there will always be a small probability for harm to occur and thus for some to argue that a specific product or technology is unsafe. In addition, scientists can many times disagree not just about safety but also how to measure it. Issues in animal toxicity studies such as the relevance of low-dose effects, of non-monotonic dose-response, of non-targeted and delayed effects, or the ways animal studies are extrapolated for indicating adverse effects in humans have not achieved a consensus among scientists.^23–28^

The presence of uncertainties can feel like an informational void. This void can be particularly problematic in emerging scientific fields. Issues like new mechanisms of action or new types of studies (e.g., long-term animal studies using whole food/feed), illustrated in the two case studies analyzed in this article, have not achieved a consensus in the wider scientific community and are thus more prone to controversies.

## 3. Methods

Two high-profile controversies around animal toxicity studies in two different fields of regulatory science (genetically modified food and food contact materials) were chosen as case studies to explore the use of the SST concept: a 2-year rat feeding study conducted with genetically modified maize by Seralini and coworkers (referred to as “Seralini study”) and a rat feeding study analyzing functional and/or morphological effects in the nervous system of pups caused by BPA maternal exposure (referred to as “Stump study”). Both studies ignited controversies around emerging science issues, emphasizing tensions between regulatory and academic science. In addition, both triggered typical responses from key stakeholders: calls for precautionary action from CSOs, requests to stick to established risk assessment requirements from industry, with regulators oscillating between the two depending on the political context. Stakeholders (CSOs or industry) selected for analysis were the main representatives of their category in each controversy.

The two case studies focus on responses to the two studies which occurred mainly in the European context.

The analysis mainly draws on documents and texts authored by the two groups of stakeholders, mainly organization websites, press releases, and peer-reviewed or non-peer-reviewed scientific publications. Fewer documents could be gathered from industry sources compared to CSO sources as industry displayed considerably less direct engagement with the controversies. In addition, the Stump study generated significantly less reaction from scientists and the public at large compared to the Seralini study.

## 4. Case Studies

### Seralini Study – GM food/feed

In 2012, a 2-year rat feeding study with herbicide-tolerant genetically modified (GM) maize conducted by a group of French researchers launched a storm in the scientific community and among the public at large. The study concluded that the GM maize developed and sold by Monsanto (NK603) and sprayed with Roundup herbicide has toxic effects including earlier death, mammary tumors, pituitary gland disabling and endocrine disruption, hepatotoxicity and nephrotoxicity and even cancer.^29^ Thereby, the study refueled an already protracted and embittered broader controversy around the safety of GM food and what studies are required to ensure a sufficient level of safety.

The study also focused attention on Roundup and its active ingredient, glyphosate, a broad-spectrum herbicide widely used in Europe and beyond. If initially it was believed to have a low toxicity for environment, for those handling it and for consumers (residues in food), recent reports seem to challenge that claim. Cuhra reviewed and analyzed published studies on the compositional analysis and animal feeding studies with glyphosate-resistant genetically modified soybeans and concluded that many crops used in pre-market animal feeding studies had not actually been sprayed with glyphosate.^30^ In addition, Cuhra signaled a potential for bias as most of the studies were conducted by, or commissioned and paid for by, industry. In addition, the International Agency for Research on Cancer (IARC), associated with WHO, classified glyphosate in 2015 as “probably carcinogenic for humans.” While this discussion is still ongoing it is worth mentioning that no regulatory agency has found glyphosate to be conclusively carcinogenic by the date of the writing of this article.

Discussions of the Seralini study included both scientific and nonscientific issues, such as the overall study design, the methods and rat strain used, and the statistical analysis performed, but also the initial denial of the study authors to grant access to raw data, and the affiliation of coauthors of the study with CRIIGEN, an CSO known to have a very critical stance to GMOs.^31–33^ Following an eruption of strong criticism in the scientific domain, the publisher of the journal in which the article first appeared, Elsevier, decided to withdraw the article. This was justified by the low number of rats used in the study (10 per group), the rat strain used (Sprague-Dawley), and the lack of links between the incidence of tumors overall to the NK603 maize or glyphosate.^34^ Elsevier also stated that no fraud or intentional misinterpretation of the data was found, and praised the corresponding author for his willingness and openness to participate in the editor’s investigation.^34^ Krimsky’s discussion of this case notes a “lack of definitive results,” but also questioned the justification for retraction as “Monsanto-funded studies using similar strains and numbers of mice were not retracted because of deficient methods.”^35^ Supporters of the conclusions in the Seralini study argued that the number of test animals was justifiable according to the goals of the study and the OECD guidelines at the time the experiment began. Also, they claimed that industry supporting the safety of GM maize used a similar number of animals for histopathologic and biochemical analyses.^33^ Seralini and his team republished their findings in 2014 in another journal, with slight changes from the original and with full disclosure of their raw data.^36^

This 2-year feeding study was published shortly after the 90-days feeding studies became mandatory for risk assessment of GM food/feed in the EU. The study results were cited by some as an indication that long-term feeding studies (beyond 90-days) are needed to eventually detect possible negative health effects for all GM food and feed.^30,37^

### CRIIGEN

CRIIGEN^a^ is an international advocacy group funded by member’s contributions, public donations, product sales and research contracts.^38^ It focuses on impacts of GM technology with a declared interest ”to expose the inadequacies of our current assessment system […] mainly in relation to public health which makes it possible for dangerous products to continue to remain unjustifiably on the market”.^39^ CRIIGEN frequently highlights – in their view – shortcomings of the established risk assessment systems and critics practices of commercialization by companies such as Monsanto. This would justify a more precautionary approach including a widening of the range of studies performed to include long-term animal feeding studies and more animal species:

- Presenting the health risk assessment for GMOs as riddled with conflicts of interests indicating possible bias as well as confidentiality issues and censorship making it very difficult and sometimes impossible to scrutinize the evidence presented by GMO developers^40^
- The need to give more attention to pesticide toxicity in GMO risk assessment of pesticide tolerant crops^41^
- Presenting the 90-day exposure period for animal feeding studies (established standard) as too short and not sufficient for GMO risk assessment^42^

Looking through SST lens (see Table 1), selective SST is employed, as CRIIGEN praises other studies that confirm the findings of the Seralini study while rejecting or ignoring studies criticizing it. Thus, a study of the effects of an adjuvant of glyphosate in the Roundup formulation (made by a group of researchers at the same university where the Seralini study was conducted) confirming the toxicity of such pesticides at low levels is praised.^41,43^ Results from two European Union funded studies and one French funded study,^44–47^ set to investigate the findings of the Seralini study and evaluate two varieties of GM maize, one of which is the same investigated by the Seralini study, are deemed to not be relevant for interpreting the data generated by the Seralini study because “their objectives and protocols are different.”^48^

**Table 1.** Strategic science translation types identified in the Seralini study and Stump study for CSOs and industry.

Stakeholder	Topic	Selective	Interpretive	Inaccurate	Contextualizing
CSO	Seralini study	CRIIGEN praises some studies that confirm the findings of the Seralini study and reject anything else.	The study was presented as evidence of GM maize and glyphosate/Roundup toxicity	The choice of the strain of rats used in the study, the Sprague-Dawley, is the go-to choice for studying the incidence of mammary tumors	The results of the study call for an immediate reconsideration of the regulatory framework
	Stump study	Supports the studies that have found effects, as opposed to the studies which did not		Recognizes that the study followed established international (OECD) and US scientific protocols, yet the study displays “major design faults”	The Stump study was inconclusive and that the dangers of BPA exposure require regulatory action
Industry	Seralini study	Not identified	The irrelevancy of the Seralini paper for current results and risk assessment practices for GM crops and Roundup	The study does not meet minimum acceptable standards for this type of scientific research	The study does not require any changes in GMO risk assessments
	Stump study	Evidence that support their view as “good science,” ”robust science and reliable statistical evaluation”, while opponents are using “bad science”: “false positives.”		Theories of Non-Monotonic Dose-Rose and Low-dose theory oversimplified and dismissed as false positives: “few animals,” “unusual methods,” “statistical outliers”	BPA is safe, “if used as intended”

Interpretive SST can be seen in CRIIGEN’s evaluation of the impact of their study. In one example, although the results of the Seralini study were found inconclusive by an extended peer-review and the authors themselves acknowledged its limitations,^49–51^ the study was presented as evidence of GM maize and glyphosate/Roundup toxicity: “many believe that the real reason for its retraction was that the study found evidence of serious health problems resulting from consumption of the GM crop and also of the herbicide, thereby putting Monsanto and the whole GM food and feed industry at risk.”^52^

There are also examples of inaccurate SST. In one such example, CRIIGEN states that the choice of the strain of rats used in their study, the Sprague-Dawley, is the go-to choice for studying the incidence of mammary tumors (a major finding of the study was that the GM maize and the pesticide Roundup cause a higher incidence of mammary tumors).^48^ However, scientists pointed out as early as 1970s that Sprague-Dawley variety has a spontaneous mammary tumor incidence that can vary considerably (5% to 90%) depending on the type of diet, caloric intake, environment and source of the particular stock making is unsuitable for the study of such tumors.^53–57^

Yet outside of specific instances of SST as described by Cordner, a ‘translation’ of scientific evidence also needs to be able to summarize the essence of the overall meaning, offer an all-encompassing and decisive view while taking the new set of data out of the wider context and attributing it a particular place in the existing knowledge, either confirming the claims of established science or attempting to create new research approaches or even new paradigms. This tactic essentially de-contextualizes the data, oversimplifying it to justify a narrow course of action. We would call this fourth type: contextualizing SST. In the case of CRIIGEN, they claim that the results of the study call for an immediate reconsideration of the regulatory framework: “Consequently, the marketing authorizations for these products must be immediately reviewed, the tests currently in force for 90 days must be extended to 2 years for all GMOs, pesticides must be tested for 2 years in low doses and in formulations, the regulatory tests of the companies must be immediately made public, and submitted to impartial expertise. In the future, they must be produced independently from the manufacturers.”^48^ (authors’ translation from French)

### Monsanto

The main industry actor in this controversy, Monsanto, is a multi-national seed and pesticide production company which has become the archetypal target of environmentalist anti-GMO groups in Europe and beyond.

In the Seralini controversy Monsanto generally rallied behind regulatory agencies, international organizations and academic researchers criticizing how the Seralini study was conducted and interpreted and what conclusions were drawn and denying their generic relevance for the safety of GM crops. The main criticisms attempt almost the same critique strategies as CRIIGEN, albeit in the opposite direction. For example, in one of their rare direct public statements, the company declared the Seralini paper inadequate for risk assessments: “The study did not meet minimum acceptable standards for this type of scientific research, the findings were not supported by the data presented, and the conclusions were not relevant for the purpose of [GMO] safety assessment.”^58^

Monsanto took a back seat to the controversy, refraining from making almost any public comments about it, even though CRIIGEN accused it to have attempted to discredit the paper. This accusation remained unproven until 2017 when the law firm Baum Hedlund Aristei & Goldman, representing U.S. residents in a lawsuit against Monsanto claiming Roundup of causing non-Hodgkin lymphoma, obtained from a US judge the permission to declassify Monsanto internal documents. Among internal e-mails, it was disclosed that Monsanto engaged scientists in efforts to get the Seralini paper retracted, ghost-wrote a pro-GMO paper on Forbes, and made efforts to make sure the campaign to retract the Seralini study would not be traced back to them.^59,60^

Similar to CRIIGEN, Monsanto’s strategies to describe findings to support a particular stance can be considered as interpretive type SST: in this case the irrelevancy of the Seralini paper for established risk assessment practices for GM crops and Roundup. The short paragraph above manages to encapsulate two types of SST. Aside from the just mentioned interpretive SST, we can find inaccurate SST. Monsanto stated that “[t]he study did not meet minimum acceptable standards for this type of scientific research.”^58^ Yet the declared aim of one of the EU-funded projects mentioned above, was to develop “guidance for the risk assessment of food and feed containing, consisting or produced from genetically modified (GM) plants as well as guidance on conducting repeated-dose 90-day oral toxicity study in rodents on whole food/feed” because “*there are no standardized protocols to study the potential short-, medium- and/or long-term toxicity of GM plants and derived products*” (added emphasis).^61^ In Monsanto’s case the contextualizing view over the Seralini controversy communicates that no changes of GMO risk assessments are required since the study does not alter the conclusion that this GM maize event is safe based on the overall weight of existing evidence.

### Stump Study – Bisphenol-A (BPA) in Food Packaging Materials

BPA is a chemical used to make a wide array of clear plastics, from DVDs to medical devices and food packaging. Soon after its first synthetization, researchers also discovered that BPA has estrogenic properties.^62^ It took several decades, however, to reveal that it can leak from containers and can act as an endocrine disruptor.^63^ Yet characterizing this adverse activity proved challenging. No only there are no adequate standardized test methods to identify such possible effects,^64^ but its biological mode of action is still under discussion. BPA, as other substances in this class of chemicals, has low acute toxicity and does not bioaccumulate, but mimics endocrine hormones at both low doses and high doses, with detrimental effects on brain, behavior, and prostate glands in fetuses, infants, and young children, sometimes even linked to carcinogenic effects as shown by animal studies.^65^ The impacts of low-dose effect reached a widespread consensus among endocrinologists, who ask for different regulatory testing on BPA,^64^ but remain contentious among some other scientists and regulatory authorities.^65–67^

In 2009 the Polycarbonate/BPA Global Group of the American Chemistry Council, an industry trade association for American chemical companies, commissioned a rat feeding study to address a disagreement over interpretation of the by then available neurotoxicity data.^68^ The study, which was compliant with Good Laboratory Practice principles and OECD Test Guidelines, searched for functional and/or morphological effects in the nervous system of pups caused by BPA maternal exposure.^69^ As expected by the authors, the exposure of pregnant rats to the highest concentrations of BPA had harmful effects in adults and their offspring, and signs of adverse effects on neurodevelopment were recorded in six pups exhibiting convulsions and seizures. However, the incidence of these observations was not statistically significant and the animals did not differ from controls in other end-points. Also, no such effects were recorded in a follow-up study, Stump et al. therefore concluded that there was no evidence that BPA is a developmental neurotoxicant in rats. The study also claimed that they could not find any low-dose effects.^69^

A subsequent review of the study by the National Food Institute at the Technical University of Denmark (acting as a scientific adviser to the Danish government) raised uncertainties about the impact on learning capacity from exposure at low doses of BPA.^70,71^ Based on this assessment Denmark invoked the precautionary principle and introduced a ban on BPA in materials that come in contact with food for children under the age of three.^72^ Reviewing the Stump et al. study and other recent literature, the French Food Safety Agency (AFSSA) concluded that there are warning signals at doses lower than the established no effect dose.^73^ AFSSA also criticized the guidelines used for the design of the protocols as not suitable for risk assessment of endocrine disruptors, particularly BPA. France banned baby bottles containing BPA in 2010.^72^

Following the publication of the new guideline compliant study and the Danish ban, the European Commission (EC) requested the European Food Safety Authority (EFSA) to evaluate the study by Stump et al. and the recent scientific literature relevant for the risk assessment of BPA, and to provide advice on the Danish risk assessment. Their findings show that no conclusion can be drawn from the Stump study on the effect of BPA on learning and memory behavior. In addition, the no-effect dose was considered low enough to allay any concern for the observed adverse effects on neurodevelopment. A minority opinion, however, argued that there were significant uncertainties about the validity of the no effect dose as adverse effects might occur at levels below it, and advised avoiding baby bottles made out of polycarbonate.^71^ The European Commission did not follow EFSA’s advise and invoked the precautionary principle and banned BPA in the manufacture and marketing of infant feeding bottles in 2011.^74^

Researchers such as Lemus consider that the BPA controversy in Europe was driven by eliminating a large body of studies reporting effects at low doses from policy consideration.^64^ The disagreements over risks to human health were caused by different views on reliability and relevance of studies.^67^

### CHEM Trust

In the EU the CSO CHEM Trust (Chemicals Health and Environment Monitoring Trust) is a key actor in the debate on endocrine disruptors. Their aim is “to prevent man-made chemicals from causing long term damage to wildlife or humans by ensuring that chemicals which cause such harm are substituted with safer alternatives.”^75^ Based in the UK, it is financed by charity foundations and was set up with seed-funding by WWF-UK.^b^ The CSO is an accredited stakeholder at the European Chemicals Agency, and also member of the UK Chemicals Stakeholder Forum, of the EDC (Endocrine Disrupting Chemicals) Free Europe coalition, and of the European Environment Bureau.^75^

CHEM Trust’s stand on BPA is “that exposure reduction is long overdue and that regulations should be put in place to try to eliminate exposure to BPA, particularly for pregnant women and children.”^76^ Although this stance seems to take for granted the long-term toxicity of BPA, the representation of the scientific evidence also discusses the presence of doubt: “A large number of animal studies, but not all, have reported effects at low doses of BPA, that lead to serum levels similar to those found in the general population. The possibility that BPA may damage human health cannot be dismissed, although regulatory assessments have noted some limitations in the low dose studies.”^76^ The organization advocates the use of the precautionary principle and reduce children’s exposure to BPA as, the claim, the scientific and regulatory assessments indicate insufficient evidence.^76^ CHEM Trust also criticized allegedly unethical behavior in regulatory assessments: “Representatives of regulatory agencies have noted the heavy lobbying by industry, and indeed there is some indication that past decisions may have been unduly influenced by economic considerations.”^76^

Regarding the Stump case, CHEM Trust is unconvinced by the evidence the study has produced but did not give it special attention on its website, except for one position paper. Although this paper recognizes that the study followed established international (OECD) and US scientific protocols, in the view of CHEM Trust the study displays “major design faults” (inaccurate SST): “These workers used the study procedure laid down in Organisation for Economic Co-operation and Development (OECD) and US Environmental Protection Agency (EPA) guidelines. […]. Moreover, experts reviewing this publication for EFSA noted that due to major design faults in this industry study by Stump et al., no conclusion could in fact be drawn about the effect of BPA on learning and memory behaviour.”^76^ The position paper further proceeds to be SST selective as it supports the studies that have found effects (without going into experimental data and protocols details), as opposed to the Stump study which did not: “Given that numerous studies have indeed found effects, CHEM Trust considers that it is not possible to be confident about the safety of BPA with regard to its effects on the brain.”^76^

In the contextualizing SST view, the essence of the story for CHEM Trust is that the Stump study was inconclusive and that the risks of BPA exposure require regulatory action.

### Bisphenol-A Europe

The industry association Bisphenol-A Europe represents the interests of the main BPA and polycarbonate producers in Europe: Covestro, SABIC, Trinseo, Hexion, and Olin.^77^ Their webpage does not have any posts about the Stump study but they do defend strongly the position that BPA was not proven to have harmful effects: “The […] EFSA, the U.S. Food and Drug Administration (FDA), the National Health and Family Planning Commission of the People’s Republic of China (NHFPC), the Japanese Ministry of Health, Labour and Welfare, Health Canada, the WHO and many more regulatory agencies worldwide concluded, based on the weight of the large amount of scientific evidence, that there is no human health concern from this chemical intermediate”; yet still using the caveat “if used as intended.”^78^

Delegitimization of studies indicating the opposite can be observed: “Regarding any studies that demonstrated opposite conclusions, experimental data often contained errors. Conclusions are often based on possible statistical mistakes rather than on actual biological phenomena.”^79^ This is a clear example of both selective and interpretive SST presenting evidence that support their view as “good science”: “robust science and reliable statistical evaluation,” and the opposite as “bad science”: “false positives.” Inaccurate SST can be identified as well, with declared “complex theories” of Non-Monotonic Dose-Rose and Low-dose theory oversimplified and dismissed as “false positives”: “few animals,” “unusual methods,” “statistical outliers.”^79^ The essence of the story for Bisphenol A Europe is that BPA is safe, “if used as intended.”

## 5. Discussion and Conclusion

Tracing the translation of science into the policy world is particularly challenging when the scientific knowledge in question is associated with considerable uncertainties. Sometimes doubts are raised by highlighting, exacerbating or even creating such uncertainties. Beyond these lessons from earlier controversies on scientific evidence in environmental and health regulatory contexts there are also other aspects to consider.^10^ The paper illustrates how the concept of SST can be used for investigating more broadly how the scientific evidence on a topic, including – in the case of animal feeding studies for toxicity assessment – a study’s raw data, historical data and accepted methodologies and theories, is strategically translated by different stakeholders.

In both case studies and for both stakeholder groups, CSOs and industry, we were able to identify all three types of SST described by Cordner: selective, interpretative and inaccurate with selective and interpretative being the most widely used (summarized in Table 1).

Uncertainties, knowledge gaps and differences in the interpretation of study results, normal occurrences in the process of knowledge production, are even more prevalent in emerging science. We argued that the long-term feeding studies with whole food diets for GMOs including pesticides used, or endocrine impacts of BPA can be considered as emerging science because their design, conduct, analysis and interpretation, and relevance for human health risk assessment has not yet been fully clarified. Thus, the process of negotiation and compromise on how to choose between different interpretations of the results is complicated by the high uncertainties associated with emerging science.^80^

In these cases, uncertainties are not just limitations of science in-the-making, but uncharted territories, “paradigm shifts” as Kuhn called them.^20^ They are also placed in wider contexts of scientific and political issues concerning safety profiles for the commercialization of products containing BPA or GMOs. In these cases, we believe a fourth type of SST becomes very important in advancing the goals of the actor employing this strategy. By concentrating, ‘distilling’ the message they believe the results are bringing forward, in lack of overwhelming evidence and in the course of negotiating the interpretation of results, while placing it in a wider scientific and political context, such actors are offering particular comprehensive and wide-ranging perspectives of the contested results. We suggest to call this fourth type of SST, ‘contextualising’ SST (Table 1). While this type of SST can exist in all cases of contested science, we believe it is particularly important in emerging science because it is most efficient in bringing other stakeholders, especially the public, into the process of negotiation and compromise that choses between different alternative interpretations of results. It does so by leaving out of the discussion portions of the wider background, being it scientific, commercial or political. While the selective SST is cherry picking the scientific information to sustain a narrow claim, the contextualizing SST chooses in which part of the wider societal context to place that scientific information.

The selective, interpretive and inaccurate SST are used to convince an audience that an actor is “right” about their scientific claims, either of their own research or of someone else’s. Contextualizing SST is used to push things further at the policy level: ‘because we are right, things need to take THIS particular direction.’ Both stakeholders investigated here, industry and CSOs, emphasize the policy aim associated with their point of view: either presenting studies as entirely *inadequate* to impact risk assessment and regulation, or as *definite proof* to transform risk assessment and overall regulation. Besides criticism, delegitimization is a major goal of both groups to discard each other’s campaigns. But while industry still promotes safety as defined by established regulatory approaches and standards for both GMO and BPA studies, CSOs are questioning and redefining those approaches and standards by referring to the precautionary principle. Both industry and CSOs attempt to challenge the credibility of each other’s claims.

Governmental agencies tend to be conservative in changing their practices in light of new scientific insights or methods. When such challenging studies emerge, agencies can decide to do nothing, be precautionary and announce bans, or conduct more research. Lack of formal acknowledgment of the limitations of the emerging scientific fields, as well as of different research approaches between regulatory and academic research contribute to the continuation of controversies in the public domain, as broader publics cannot easily assess the relevance of the new scientific evidence presented. When a study is not clearly accepted nor rejected it creates confusion and even ground for conspiracy theories. As a result, depending on the political climate, regulatory agencies can be forced to be precautionary, as in the case of BPA, or conduct more research, as in the case of GM maize.

New data which differ significantly from evidence published earlier, can also indicate issues previously overlooked or investigative errors that need to be better understood in order to be avoided. Thus, rather than being downplayed or overly highlighted such data deserve scrutiny and may trigger further research.

Furthermore, while Cordner does not differentiate between established and emerging science, the SST concept acknowledges the importance of uncertainties in science as enablers and reasons of existence of various types of SST. Yet we believe this can be taken further as SST can be a powerful concept to guide further investigations how scientific information can be manipulated in very complex emerging science controversies. The informational void and thus potential for information manipulation can be higher than for established science. In particular, identifying a fourth type of SST, the ‘contextualising’ SST, can help identify the types of arguments or goals different actors are most likely to use.

## Notes

^1.^
The online search that gathered these results was done in 2018. At the time, CRIIGEN maintained an English version of their website. As of early 2020 the site is only in French and is completely reorganized.

^2.^
The Esmée Fairbairn Foundation, the Waterloo Foundation, The Polden-Puckham Foundation, The European Environment & Health Initiative, The Orp Foundation, previously by The Garfield Weston Foundation, The Ashden Trust, Greenpeace Environmental Trust (CHEM Trust 2010).

## Statements and Declarations

This paper summarises the results of research activities considerably extending on what was found in the course of a social science task in the course of the project “GMP Two Year Safety Testing” (G-TwYST). G-TWYST was funded by the European Union’s Seventh Framework Programme (Grant No 632165) completed in 2018. This project conducted a model long-term animal feeding study with herbicide tolerant genetically modified maize and elaborated guidance on the scientific quality criteria and conclusions on the added value of such studies for GMO risk assessment. The overall aim of this task was to gain a better understanding of the issues and the dynamics of the disputes on animal feeding studies, in particular of the interplay of scientific facts, values, world views and interests. The publication itself received funding from TU Graz Open Access Publishing Funds.
